# Pathology of Decay

**Published:** 1875-02

**Authors:** J. B. Wheeler

**Affiliations:** Lawrence


### PATHOLOGY OF DECAY.

BY J. B. WHEELER, OF LAWRENCE.

*Read before the Kansas State Dental Society, May 5th,* 1875.

In writing on the pathology of dental caries, we at once recognize a subject which is fit for a volume instead of a single essay or paper, and one which our ablest and best writers have discussed for nearly a century, and seem still inclined to present new theories and refute old ones, till it seems hardly possible to bring up anything new on the subject, and if it were possible the propriety might be questioned. We have had almost daily new and startling theories thrown before us of almost every variety of which a vivid imagination is capable, and still there is in the minds of the greater number of practitioners of the present day but a misty conception of the cause of caries of the human teeth. That we have made progress as specialists cannot be denied, but have we, as a class given the cause of the diseases which we treat, that attention and patient investigation which they deserve,and which it is our duty to do, or, have we been content to merely follow the mechanical part, simply treating such cases as occur in the rounds of daily practice, without thought or knowledge of the cause of such service being required? To the operator, devoting his time exclusively to the laboratory, and the daily rounds of active practice, it is not easy to see the intimate connection between general pathology and gold foil; but there can no longer remain a doubt, that upon a knowledge of the former most certainly depends much of the usefulness of the latter. Promptness in diagnosing, confidence in prognosing, and a course of treatment which the sequel will show to have been based upon knowledge and judgment, will give to the patient a feeling of entire confidence, and are the instigators of that feeling of self-satisfaction which is after all the fee of fees.

In the early history of dentistry we find very little of present value on pathology, and I think we are safe in saying that to Robert Arthur, in 1853, belongs the credit of the first work on dental pathology which can be termed scientific, and in 1869 Garretson made a valuable addition, in giving to both the medical and dental professions his excellent work on "diseases and surgery of the month, jaws and associate parts." While, at present, there are being printed in the Dental Cosmos, a series of papers on dental pathology and therapeutics from the able pen of Dr. Flagg,. which are well worth careful study by the students and members of the profession.

After reviewing and noting progress made in the last twenty years, we readily discover a great stride in dental pathology from the ancient observers, and arrive at an era in which it is strangely interesting to make quotations from old writers, such as "the cause of tooth-ache is only known to God, and the cause of decay of the teeth is beyond the comprehension of man." Such questions as these, I am happy to state, are of the past and the present presents to us plausible theories on which to treat and prevent those abnormal conditions of the teeth, the causes of which were formerly supposed to be known only to the deity.

In dental caries, pathologically speaking, vital force plays an important part, and as we trace changes or periods of decay of the teeth from childhood to old age, how markedly we see the extent and amount of the one keeps pace as it were with the state of the other. Thus we see in those cases of prolonged fevers especially those of typhoid nature, the immense strides which caries almost always make. No doubt a great amount of caries in the teeth of young patients is due to the lack of vital force, which is brought about by our improved modes of living, the tendency of man in the last half century being to attain to respectability, or to that position in which he can live with the least exertion, and rear his children in comparative luxury, which is the precursor of a diminished or low state of vitality. Assuming that the teeth are a part of the vital economy, and necessarily under the domination of vital influence, we must recognize that all influences, tending to diminish vital action, must, affect first and most seriously such portions of the organism as are not absolutely essential to vitality, and as want of vital force at the time of formation and eruption of the teeth is felt, need we wonder at the immediate decay presenting itself in the teeth of this class of patients. From the time of the presentation of permanent teeth to about the twentieth year is conceded to be the most critical period which the denture of the comparatively healthy patient undergoes, on account of the constant draughts on vitality which go to give the patient growth, after the comparative completion of which there is usually a marked cessation of dental caries, and also a general strengthening of the vital system. If the teeth are carried to the age of manhood, with perfect formation, and free from caries there may be expected comparative immunity from disease in after life. For many years it has been extensively conceded that the ordinary changes of temperature, as those to which the teeth are subjected in the ordinary food taking of civilized life, were the concomitants of immediate decay. That they are prejudicial to the welfare of the teeth, there can be no doubt; but their influence is beyond question indirect. The chemical theory of decay seems at once the most tangible of any which has been explained, since in almost every case of caries, experiment shows an acid condition of decomposing tissue, and this condition changing in proportion to the greater or lesser rapidity of diseased action finally diminishing in those cases of slow decay so as to be hardly perceptible.

This chemical change in active caries, is somewhat singular and interesting, Flagg says concerning it, "There is a decided difference which we find to exist between the ordinary chemical action of acids upon such salts and organic tissue as compose tooth structure and that action which results in caries, for it seems no less than entirely different. Frequent and long continued experimentation has failed, in almost every instance, to produce any result which could be considered as analagous to caries."

The direct action of acids coming in contact with teeth in the mouth produces a different result from true caries, as we often observe from the too free use of lemons, and the use of acid medicines often soften the enamel to that degree that it can be easily cut or scraped away, while after this operation leaving the tooth intact, and comparatively free from the dangers of caries, if properly polished, and the use of acids discontinued. The parasitic predisposing cause of caries is one on which there are at present many theories, and, beyond doubt, patient investigation and the microscope will accomplish wonders in this direction. But as yet there has been very little save startling announcements and gigantic theories, without the patient inquiry and research to maintain the grounds taken.

Treatment not coming under the head of pathology, I only wish to remark that the time is surely about to dawn when more general and constitutioual means are to be employed to prevent caries than are now taken by the majority of dentists, and a time when to prevent an operation will be more appreciated by our patrons than at present are our most beautiful efforts.

